# Characterization of the interactions between Codanin-1 and C15Orf41, two proteins implicated in congenital dyserythropoietic anemia type I disease

**DOI:** 10.1186/s12860-020-00258-1

**Published:** 2020-03-23

**Authors:** Grace Swickley, Yehoshua Bloch, Lidor Malka, Adi Meiri, Sharon Noy-Lotan, Amiel Yanai, Hannah Tamary, Benny Motro

**Affiliations:** 1 The Mina and Everard Goodman faculty of life sciences Bar-Ilan University, 52900 Ramat-Gan, Israel; 2 Hematology/Oncology Department, Schneider Children’s Medical Center of Israel, Petach Tikva, Israel; 3 Felsenstain Medical Research Center, Sackler School of Medicine, Tel Aviv University, Tel Aviv, Israel

**Keywords:** Congenital dyserythropoietic anemia type I, C15Orf41, Phylogenetic profiling, CNOT1

## Abstract

**Background:**

Congenital dyserythropoietic anemia type I (CDA I), is an autosomal recessive disease with macrocytic anemia in which erythroid precursors in the bone marrow exhibit pathognomonic abnormalities including spongy heterochromatin and chromatin bridges. We have shown previously that the gene mutated in CDA I encodes Codanin-1, a ubiquitously expressed and evolutionarily conserved large protein. Recently, an additional etiologic factor for CDA I was reported, C15Orf41, a predicted nuclease. Mutations in both CDAN1 and C15Orf41 genes results in very similar erythroid phenotype. However, the possible relationships between these two etiologic factors is not clear.

**Results:**

We demonstrate here that Codanin-1 and C15Orf41 bind to each other, and that Codanin-1 stabilizes C15Orf41. C15Orf41 protein is mainly nuclear and Codanin-1 overexpression shifts it to the cytoplasm. Phylogenetic analyses demonstrated that even though Codanin-1 is an essential protein in mammals, it was lost from several diverse and unrelated animal taxa. Interestingly, C15Orf41 was eliminated in the exact same animal taxa. This is an extreme case of the Phylogenetic Profiling phenomenon, which strongly suggests common pathways for these two proteins. Lastly, as the 3D structure is more conserved through evolution than the protein sequence, we have used the Phyre2 alignment program to find structurally homologous proteins. We found that Codanin-1 is highly similar to CNOT1, a conserved protein which serves as a scaffold for proteins involved in mRNA stability and transcriptional control.

**Conclusions:**

The physical interaction and the stabilization of C15Orf41 by Codanin-1, combined with the phylogenetic co-existence and co-loss of these two proteins during evolution, suggest that the major function of the presumptive scaffold protein, Codanin-1, is to regulate C15Orf41 activities. The similarity between Codanin-1 and CNOT1 suggest that Codanin-1 is involved in RNA metabolism and activity, and opens up a new avenue for the study of the molecular pathways affected in CDAI.

## Background

Congenital dyserythropoietic anemias (CDAs) are a heterogeneous group of inherited disorders sharing the common feature of impaired erythropoiesis and characteristic cytopathology of erythroid cells. This group of disorders has been classically classified into three major types (CDA I-III) and CDA variants. CDA type II, the most common type, is a recessive disease characterized by bi/multinucleated erythroblasts with marginal cisternae. So far only one causative gene for CDA II has been described, SEC23B, a core component of the coat protein-complex II [1, 2]. As the coat proteins serve essential roles in all tissues and cells, the reason for the erythroid-specific phenotypes is not clear. It has recently been suggested that in human (but not in mouse) SEC23B is highly expressed in the erythroid system and thus uniquely affects this system [3]. The key CDA type III characteristic is giant multinucleate erythroblasts with up to 12 nuclei per cell. CDA type III is a dominant disorder caused by a specific mutation in kinesin family member 23 (KIF23) [4]. KIF23 is a key regulator of cytokinesis, and its deficiency in HeLa cells resulted in bi- and multi-nuclei, similar to the clinical hallmark of CDA III erythroblasts [5]. CDA variants do not fulfill classical bone marrow morphological or biochemical criteria. In line with the erythroid-specific phenotypes, the variants recently designated CDA type IV was reported to be caused by a monoallelic mutations in the erythroid transcription factors KLF1 or GATA1 [6].

CDA type I is a rare recessive disorder characterized by binucleate macrocytic erythroblasts and internuclear bridges. Bone marrow electron microscopy shows a spongy (“Swiss cheese”) appearance of the heterochromatin in erythroblasts. Using positional cloning in affected Bedouin families, it has been established that the mutated gene is *CDAN1*, encoding Codanin-1 protein [7]. Human Codanin-1 is a 1227 amino acid long protein with no apparent enzymatic activity, and has no apparent paralogous protein relative. More than 50 *CDAN1* causative mutations have been reported [8] and none of the patients appear to be homozygous for a null type *CDAN1* mutation, suggesting that a complete lack of Codanin-1 protein may be embryonic lethal [7, 9, 10]. In agreement, mice homozygotes for a null *Cdan* allele die at an early stage of embryonic development (i.e. ~ 7 dpc), before the onset of erythropoiesis [11]. Recently, in a Cas9/sgRNA screen for genes essential for the survival of two human cancer cell lines, *CDAN1* was shown to be a vital gene [12]. Mutation in the *Drosophila* homolog of CDAN1, discs lost (dlt), has been described [13]. Mutant cells for dlt were able to proliferate, but had a pronounced growth disadvantage, and dlt was found to be involved in the survival of differentiated cells. Recently, another etiologic factor for CDA I was reported [14], designated C15Orf41. The function of the protein is not known, but its sequence suggests that it serves as an endonuclease, presumably related to Holliday junction resolvases [14].

Mutations in both CDAN1 and C15Orf41 genes results in very similar erythroid phenotype. However, these genes’ specificity to the erythroid lineage is not clear, and neither is the possible connection between these two etiologic factors. We demonstrate here a physical interaction between Codanin-1 and C15Orf41. Codanin-1 overexpression elevates C15Orf41 protein levels and shifts it from the nucleus to the cytoplasm. Phylogenetic analysis demonstrated that while Codanin-1 is an essential protein in mammals, several unrelated animal taxa loose the gene. Quite surprisingly, the same taxa also loose C15Orf41, strengthening the hypothesis that the two proteins participate in the same pathway. Lastly, we revealed high similarity in the 3D structure between Codanin-1 and the CNOT1 subunit of the CCR4-NOT deadenylase complex, implying similar functions.

## Results

### Codanin-1 interacts with C15Orf41 protein

**Fig. 1:**
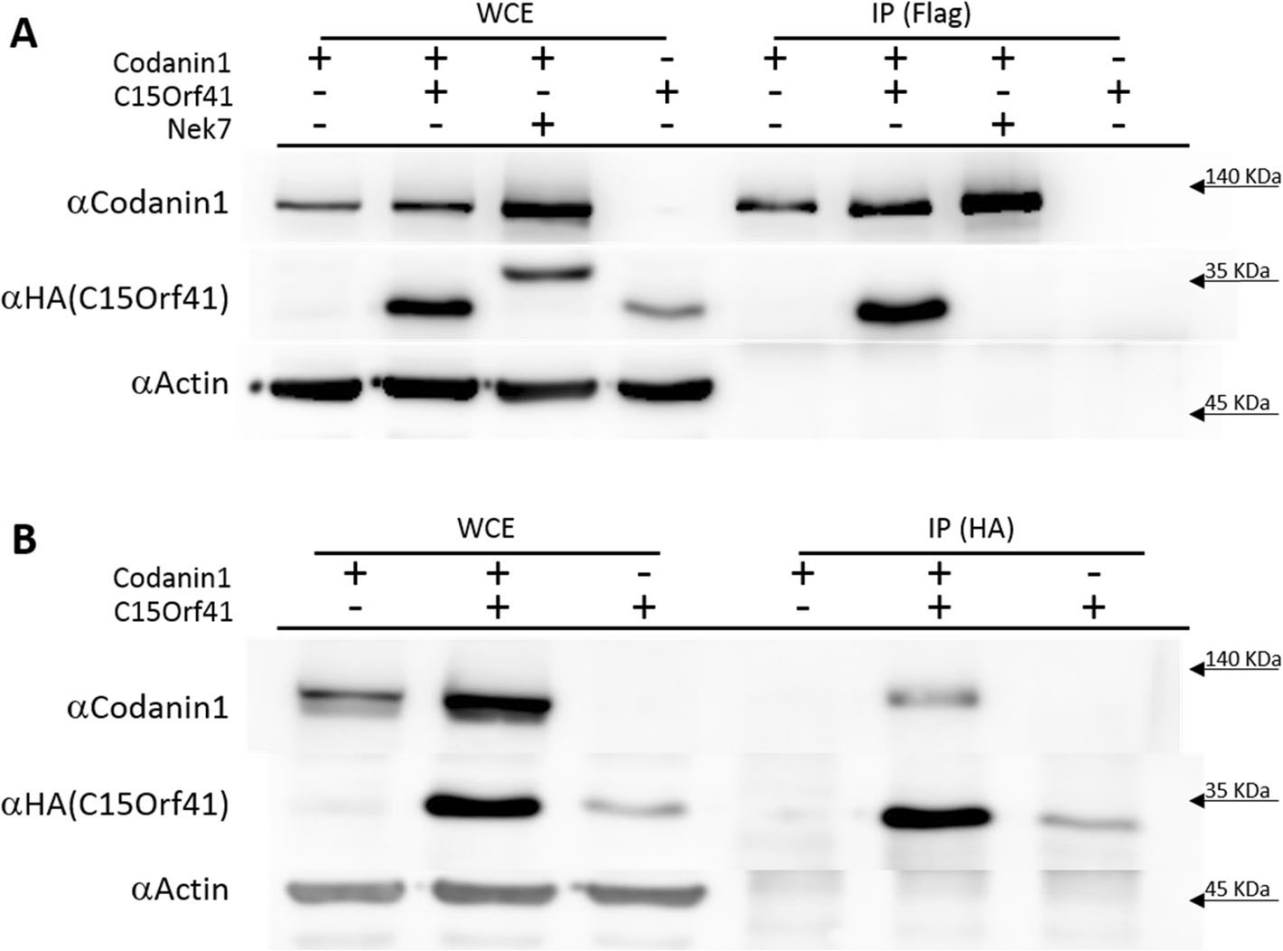
Co-immunoprecipitation of HA-C15Orf41 and Flag-Codanin-1. **a**. Hela cells were transiently co-transfected with Flag-Codanin-1 alone, HA-C15Orf41 HA-Nek7 or C15Orf41 alone, precipitated with Flag antibodies and incubated with the indicated antibodies. **b**. The reciprocal experiment - HeLa cells were transiently co-transfected with Flag-Codanin-1 alone, HA-C15Orf41 or C15Orf41 alone, precipitated with anti-rabbit HA antibodies and incubated with the indicated antibodies. WCE- Whole cell extract

**Fig. 2:**
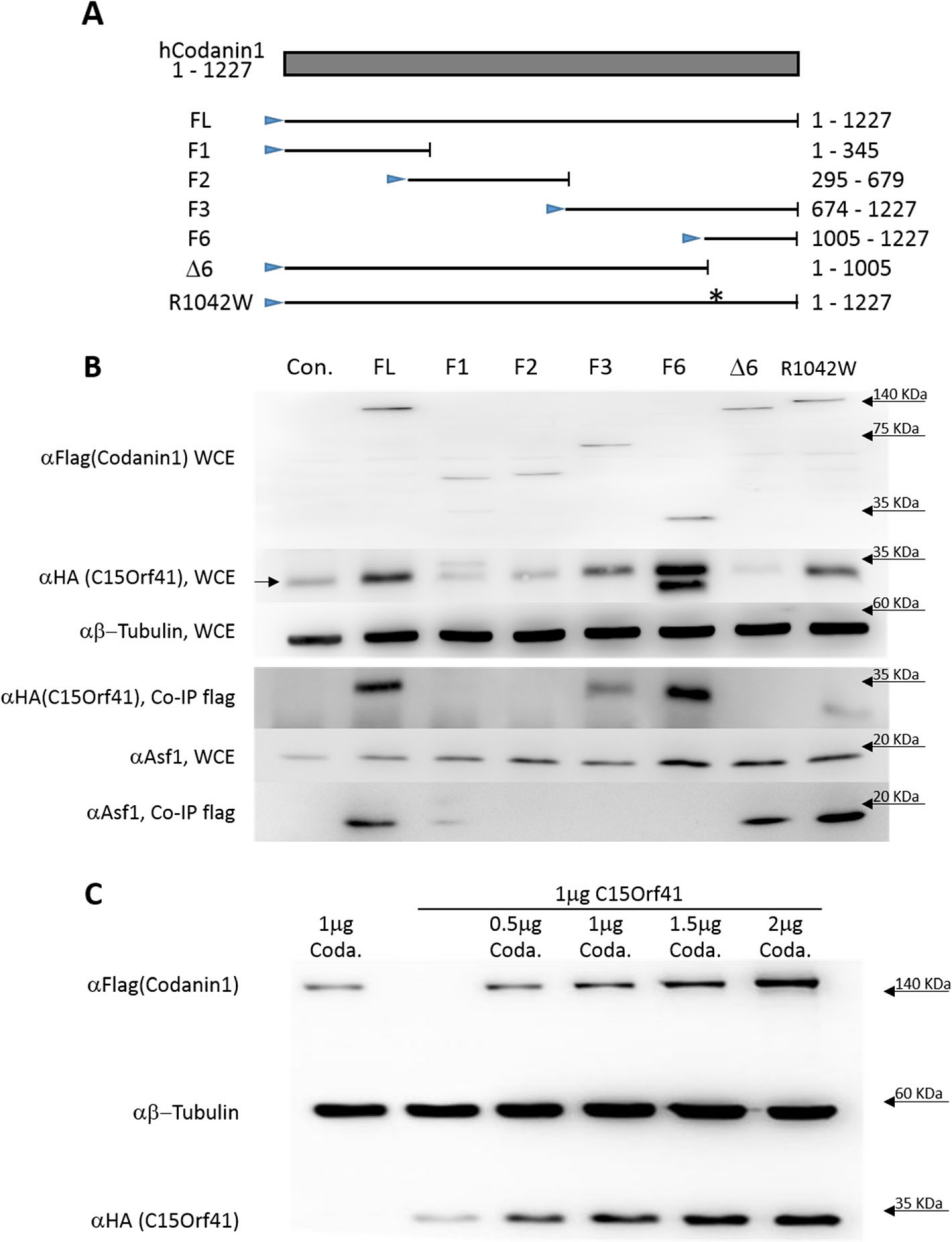
The C-terminal domain of Codanin-1 binds to C15Orf41 and elevates its levels. **a**. Schematic representation of Codanin-1 sub-fragments. The amino acid composition of each fragment is indicated to the right. FL – full-length protein. The arrowhead indicates the flag tag. **b**. Co-Immunoprecipitation of Codanin-1 fragments. HeLa cells were transfected with HA-C15orf41 and the indicated Codanin-1 fragments. Co-immunoprecipitation was performed using Flag antibody. Whole cell extracts (WCE) and co-immunoprecipitated proteins (CO-IP) are shown. Codanin-1 overexpression did not influence the levels of the endogenous ASF1 levels, and the N-terminus of Codanin-1 (F1) precipitated ASF1. **c**. C15Orf41 protein elevation is dependent on the levels of Codanin-1 overexpression. Western blot of HeLa cell lysate transfected with equivalent levels of HA-C15ORF41 and different concentrations of Codanin-1-Flag. β-tubulin was used as a loading control

To identify Codanin-1 interacting proteins, we immunoprecipitated either the endogenous Codanin-1 or overexpressed Flag-tagged Codanin-1 from human HeLa cells, and identified the interacting proteins by MS/MS analysis. One of the proteins precipitated using both approaches was C15Orf41. C15Orf41 was reported earlier as an additional etiological agent for CDA I [14]. However, physical interaction between Codanin-1 and C15Orf41 has not been demonstrated. Reciprocal co-immunoprecipitations using both tagged-Codanin-1and tagged C15Orf41 confirmed the association between C15Orf41 and Codanin-1, while the prominent protein, actin, and an unrelated protein (Nek7 kinase) did not co-precipitated (Fig. 1a, b). In order to map the Codanin-1’s region responsible for the interaction with C15Orf41, sub-fragments of Codanin-1 were created (Fig. 2a). Subsequent co-immunoprecipitation assays confirmed previous observations that the histone H3/H4 chaperone Anti-Silencing Function 1 (ASF1) binds to the N-terminus of Codanin-1 [15] (Fig. 2b). In contrast to ASF1, C15Orf41 interacted with the C-terminal 222 amino acids of Codanin-1 (a.a.1005 to 1227) (Fig. 2b). Indeed, Codanin-1 protein lacking the last 224 amino acids did not precipitate C15Orf4 (Fig. 2b). Even though the mutation in Codanin-1 protein found in the Israeli Bedouin families (R1042W) is located within this fragment, this mutation did not affect the binding to C15Orf4 (Fig. 2b).

### Codanin-1 influences C15Orf41 levels and localization

**Fig. 3:**
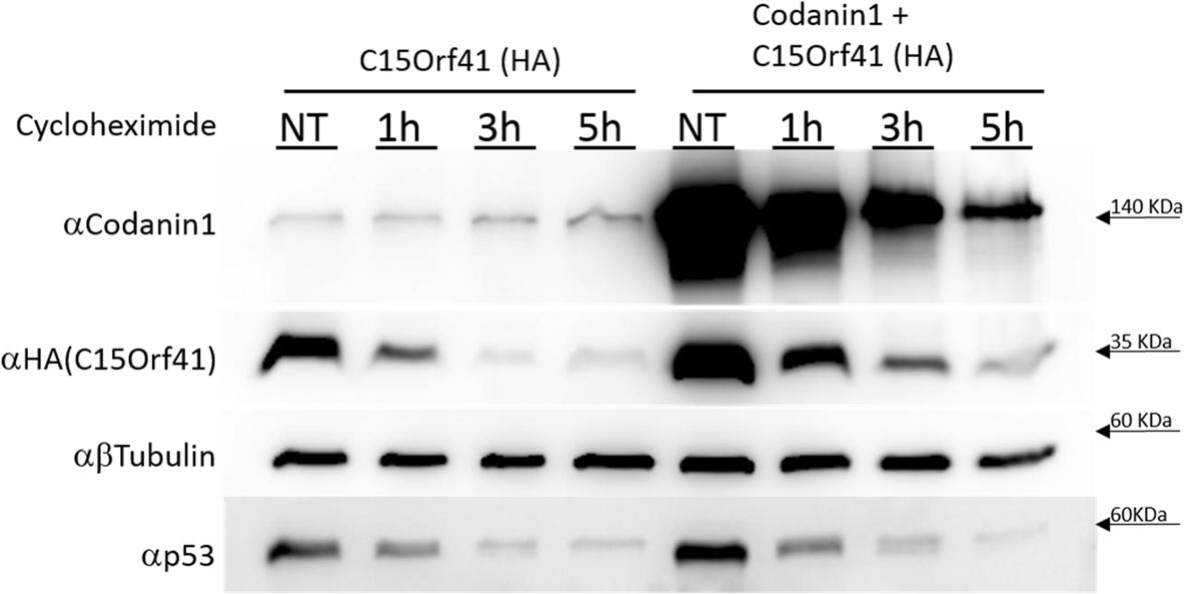
Codanin-1 lengthens C15Orf41 half-life time. Cells were treated with cycloheximide for the indicated times and were run on a western blot. β-tubulin antibody was used as a loading control. Note that the quantity of HA-C15Orf41 DNA in the co-transfection with Codanin-1 was half of the quantity transfected in HA-C15Orf41 and vector treatment in order to get more equal initial levels of HA-C15Orf41. NT not treated

**Fig. 4:**
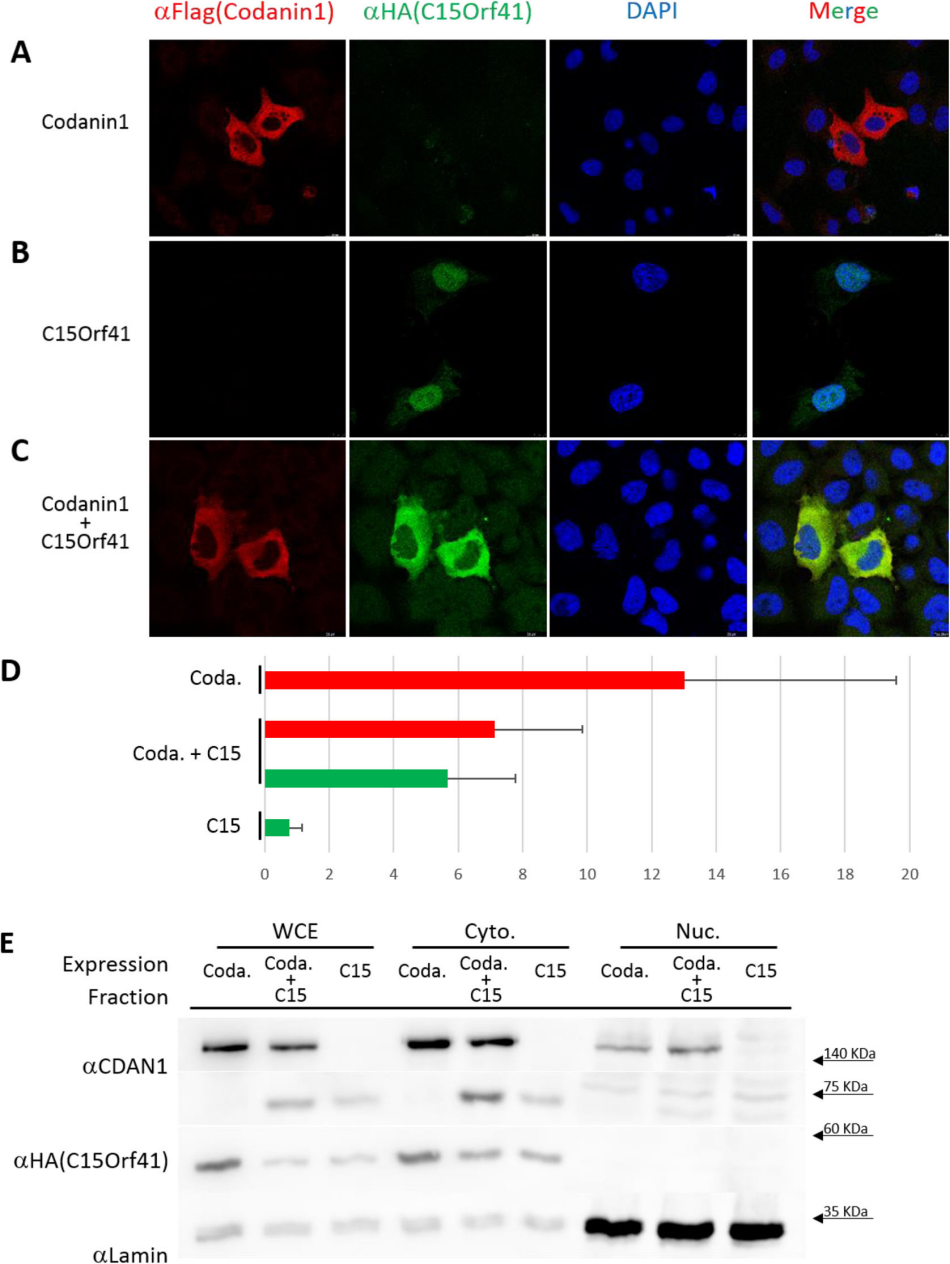
Codanin-1 overexpression shifts C15Orf41 protein from the nucleus to the cytoplasm. **a**-**c**. HeLa cells co-transfected with the indicated constructs were stained with DAPI (blue) and antibodies against HA (green) and Flag (red). The immunofluorescence visualization of the cells was performed with axioimager microscopy. **d**. Red - The ratio of Codanin-1 protein levels in the cytoplasm compared to the nucleus. The *p*-value of the ratio between Codanin-1 alone to co-transfection of Codanin-1 and C15Orf41 is 0.027. Green – The ratio of C15Orf41 protein levels in the cytoplasm compared to the nucleus. The p-value of the ratio between C15Orf41 alone to co-transfection of Codanin-1 and C15Orf41 < .00001. **e**. Cell fractionation following overexpression of C15Orf41 alone or co-expression of C15Orf41 and Codanin-1. The purity of the fractions was confirmed by probing with antibodies against A lamin (nuclear) and tubulin (cytoplasmic). Note that due to the instability of C15Orf41 when expressed alone (without Codanin-1), the levels of the transfected C15Orf41 DNA were three times higher than the transfected levels when it was co-transfected with Codanin-1

Interestingly, we noticed that upon co-transfection of C15orf41 with Codanin-1 there was a sharp elevation in the levels of C15Orf41 protein (4.7 ± 2.0 times) (Figs. 1a, b; 2b). This rise in the levels of C15Orf41 protein was codanin-1 dose-dependent (Fig. 2c). Thus. in the following experiments in which transfection of C15Orf41 alone was compared to co-transfection of C15Orf41 and Codanin-1, the levels of C15Orf41 DNA were 2–3 times higher (than the levels in the co-transfection) in order to get comparable levels of C15Orf41 protein (Figs. 3, 4). Mapping of the Codanin-1 domain responsible for this elevation suggested that it is mainly dependent of the C-terminus of Codanin-1, and the construct lacking the last 224 a.a. has much lower ability to enhance the levels of C15Orf41 levels (Fig. 2b). To discount the possibility that the enhancement is the result of co-transfection of the two constructs, we established a HeLa Tet-On cell line in which codanin-1 is expressed under the tet-responsive element (TRE), and HeLa Tet-Off cell line in which C15Orf41 is expressed under the TRE. As expected, treatment with doxycycline or its abolishment, enhanced the levels of Codanin-1 or C15Orf41, respectively. In both cases, higher levels of Codanin-1 enhanced the levels of C15Orf41, revealing that co-transfection is not needed for C15Orf41 protein elevation (Fig. S1).

We next asked whether the higher levels of C15Orf41 in the presence of Codanin-1 are the result of an effect on the half-life of the C15Orf41 protein. To this end, we treated the transfected cells with the translation inhibitor, cycloheximide (CHX). HeLa cells were transfected with C15Orf41 alone or were co-transfected with C15Orf41 and Codanin-1, followed by incubation with CHX. As can be seen in Fig. 3, C15Orf41 is an unstable protein and following 3 h of incubation with cycloheximide it almost completely disappeared. However, co-transfection of Codanin-1 resulted in extension of the half-life of C15Orf41. As a control, the half-life of P53 protein was not influenced by co-expression of Codanin-1 (Fig. 3).

One option for Codanin-1’s influence on the levels of C15Orf41 protein is by inhibiting its degradation by the proteasome. MG132 is a potent, reversible, and cell-permeable proteasome inhibitor. Thus, if Codanin-1 influences C15Orf41 protein’s levels by inhibition of its degradation by the proteasome, we would expect that in the presence of MG132, Codanin-1 will not have a significant effect on C15Orf41 levels. HeLa cells were transfected with C15Orf41, with or without Codanin-1, and were incubated with MG132 or with its solvent, DMSO. Importantly, even in the presence of MG132, high levels of Codanin-1 correlated with higher levels of C15Orf41 (Supplementary Fig. S2). This suggests that at least part of the Codanin-1 effect on C15Orf41 levels is not due to escape from the proteasome. Quite surprisingly, a decrease in the levels of both Codanin-1 and C15Orf41 were seen following the treatment with MG132 (Supplementary Fig. S2). The lower levels of C15Orf41 in the MG132 treatment could be due to the lower levels of Codanin-1. However, the lower levels of Codanin-1 are harder to explain, and are in contrast with most proteins, whose levels rise following the inhibition of the proteasome. It is thus possible that Codanin-1 is normally degraded by a pathway that is downregulated by the proteasome, and thus MG132 relieves Codanin-1’s degradation.

Codanin-1 is primarily cytoplasmic (Fig. 4a). The sequence of C15Orf41 suggests that it might serve as an endonuclease, related to restriction endonucleases. As could be surmised from this assumption, overexpressed C15Orf41 tagged with HA was observed almost exclusively in the nucleus. (Fig. 4b). However, when C15Orf41 was co-transfected with codanin-1, C15Orf41 was shifted to the cytoplasm (Fig. 4c). The ratio of the levels of C15Orf41 in the cytoplasm compared to its levels in the nucleus was 0.75 ± 0.4, while following co-expression of Codanin-1 the ratio was 5.7 ± 2.1 (*p*-value < .00001). Fractionation of HeLa cells extracts transfected with either C15Orf41 alone or C15Orf41 and Codanin-1 confirmed the enrichment of C15Orf41 in the cytoplasm following co-transfection with Codanin-1 (Fig. 4e). However, presumably due to instability of the nuclear C15Orf41 protein, only low levels of the protein were detected in nucleus (Fig. 4e).

To corroborate whether C15Orf41 and Codanin-1 binding is essential for the change in C15Orf41 localization, fragments of Codanin-1 were co-expressed with HA-C15Orf41, and C15Orf41 localization was observed by immunofluorescence. The C15Orf41 localization shift was observed with Codanin-1’s fragments 3 and 6, similarly to those which bind C15Orf41 (supplementary Fig. S3). As a control, only the N-terminal fragment of Codanin-1 influences ASF1 localization (Supplementary Fig. S4). In line with the ability of Codanin-1 R1042W mutant protein to bind C15Orf41, it also caused a similar change in the localization in C15Orf41 (not shown). Thus, Codanin-1 may serve as a scaffold protein holding C15Orf41 (and ASF1) in the cytoplasm and stabilizing C15Orf41 levels.

### Codanin-1 and C15Orf41 share very similar phylogenetic profiles

The concept of “phylogenetic profiling” assumes that two proteins which participate in the same pathway, or are part of the same structure, will have an evolutionary tendency to be eliminated or to be preserved in the same taxa [16]. We therefore examined whether Codanin-1 and C15Orf41 share similar phylogenetic distributions. Codanin-1 was first identified in human [7] and in *Drosophila* [13]. To follow its ancestral origin and phyologenetic distribution we searched for homologues of human Codanin-1 in all eukaryotic major groups using the NCBI BlastP program.

**Fig. 5:**
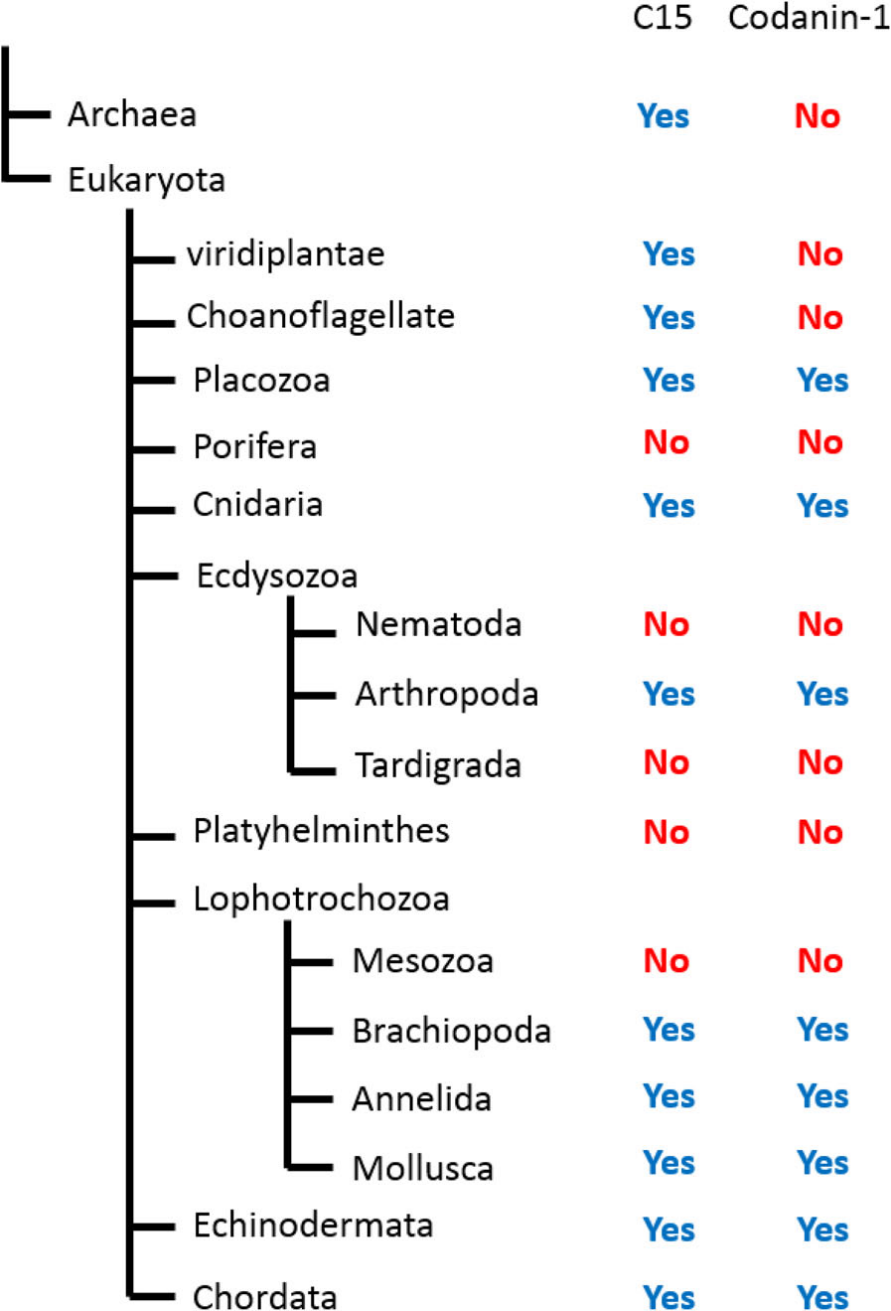
Highly similar distribution of C15Orf41 and Codanin-1 in animal taxa. The existence or absence of C15Orf41-related or Codanin-1-related proteins is indicated on the eukaryotes phylogenetic tree

Codanin-1 was found to be quite highly conserved during metazoan evolution. Codanin-1 orthologues are present in most animals (including the ancestral Placozoa and Cnidaria). However, even though it is an essential protein for mammalian development and for mammalian cell survival [12], it was apparently lost from several diverse taxa including Porifera (sponges), Nematoda, Tardigrada (“water bears”), Platyhelminthes (flat worms) and Mesozoa (worm-like parasites of marine invertebrates) (Fig. 5). In addition, there are Codanin-1 orthologues in several protists including the fungus-like oomycetes and the microalgae diatoms (both belonging to the Stramenopiles taxa), but not in choanoflagellates, the closest living protists of the animals. The presence of Codanin-1-like protein in only a few protists can suggest horizontal transfer from metazoans into these groups or (less plausibly) a massive loss in most of the protists’ groups.

In comparison, C15Orf41 roots are more ancient than those of Codanin-1, and its orthologues are already found in Euryarchaeotes, a phylum of Archaea (but not in other Archaeal phyla) (Fig. 5). In addition, it is found in several protists’ groups including those in which Codanin-1 is present (diatoms and oomycetes). Quite strikingly, no homologues of C15Orf41 are present in the animal groups Porifera, Nematoda, Tardigrada, Platyhelminthes and Mesozoa, exactly the same groups from which Codanin-1 was lost (Fig. 5). Thus, C15Orf41 exists in all of the taxa in which Codanin-1 exists (but not vice versa), and they were simultaneously lost in several unrelated animal groups.

### Codanin-1 is structurally similar to the multifunctional protein, CNOT1

**Fig. 6:**
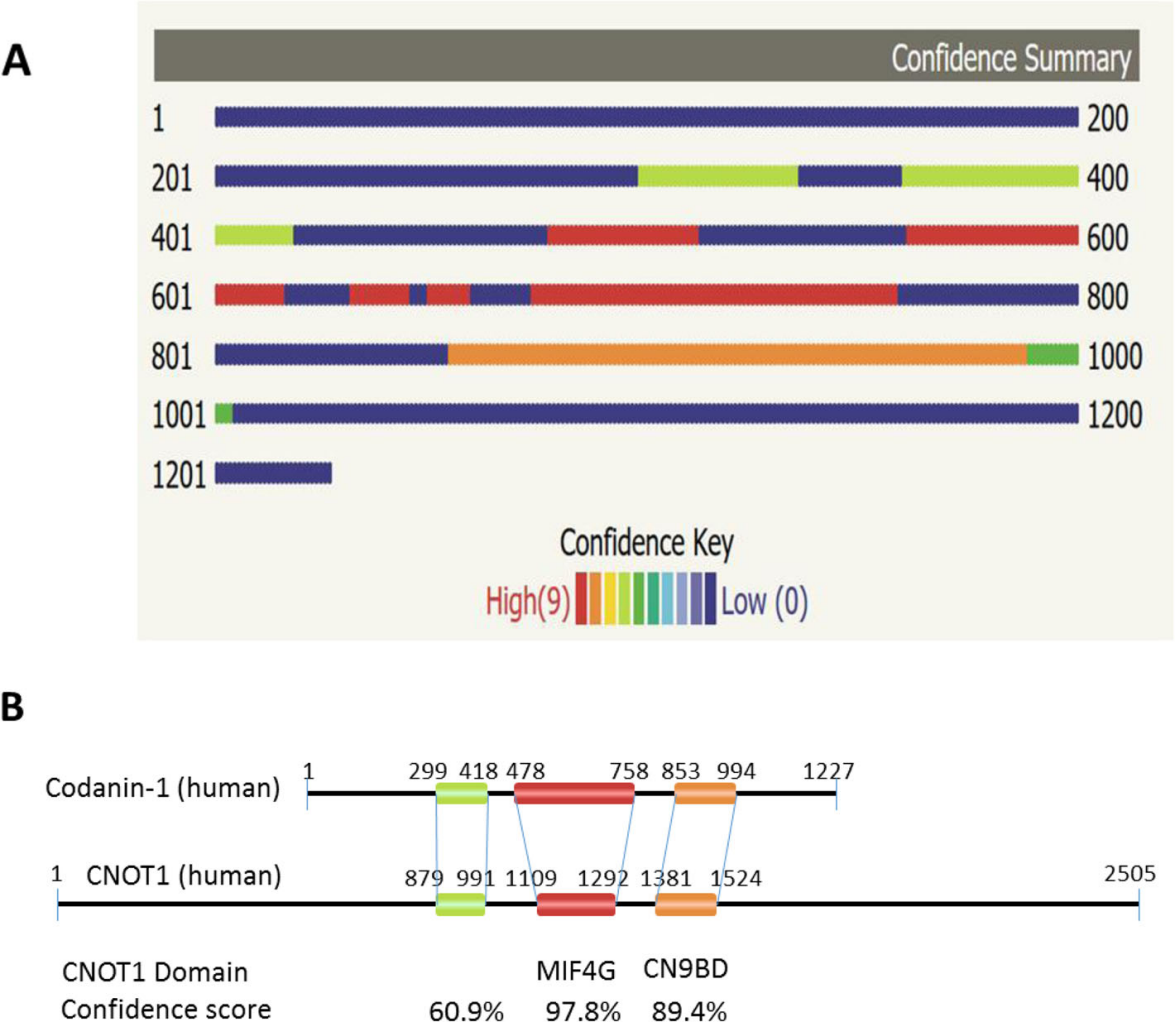
Schematic presentation of Codanin-1 domains predicted to be similar to experimentally determined 3D structures. **a**. The regions in human Codanin-1 protein predicted to be similar to pre-determined 3D structures is colored according to the degree of confidence. **b**. Alignment of the similar regions of Codanin-1 and CNOT1 proteins. The two most similar regions correspond to the MIF4G and CN9BD domains of CNOT1

The identity of the ancestral protein(s) which gave rise to Codanin- 1 is not clear, as BlastP searches do not reveal any paralogous proteins or an obvious domain [11, 17], giving no clue about Codanin-1 functions. As protein structure is more conserved in evolution than protein sequence, we have used the protein structure homology-modeling server Phyre2 to predict the 3D structure of codanin-1, and to identify model proteins which carry similar structures [18]. Interestingly, the three highest scoring templates used to model human Codanin-1 structure were all derived from a single protein, CNOT1 (Fig. 6a,b). The confidence scores were 97.8, 89.4 and 60.9% for a.a. 478–758, 855–988 and 299–418 of Codanin-1 using a.a. 1109–1292, 1381–1524 and 879–991 of human CNOT1 as a model (respectively).

**Fig. 7:**
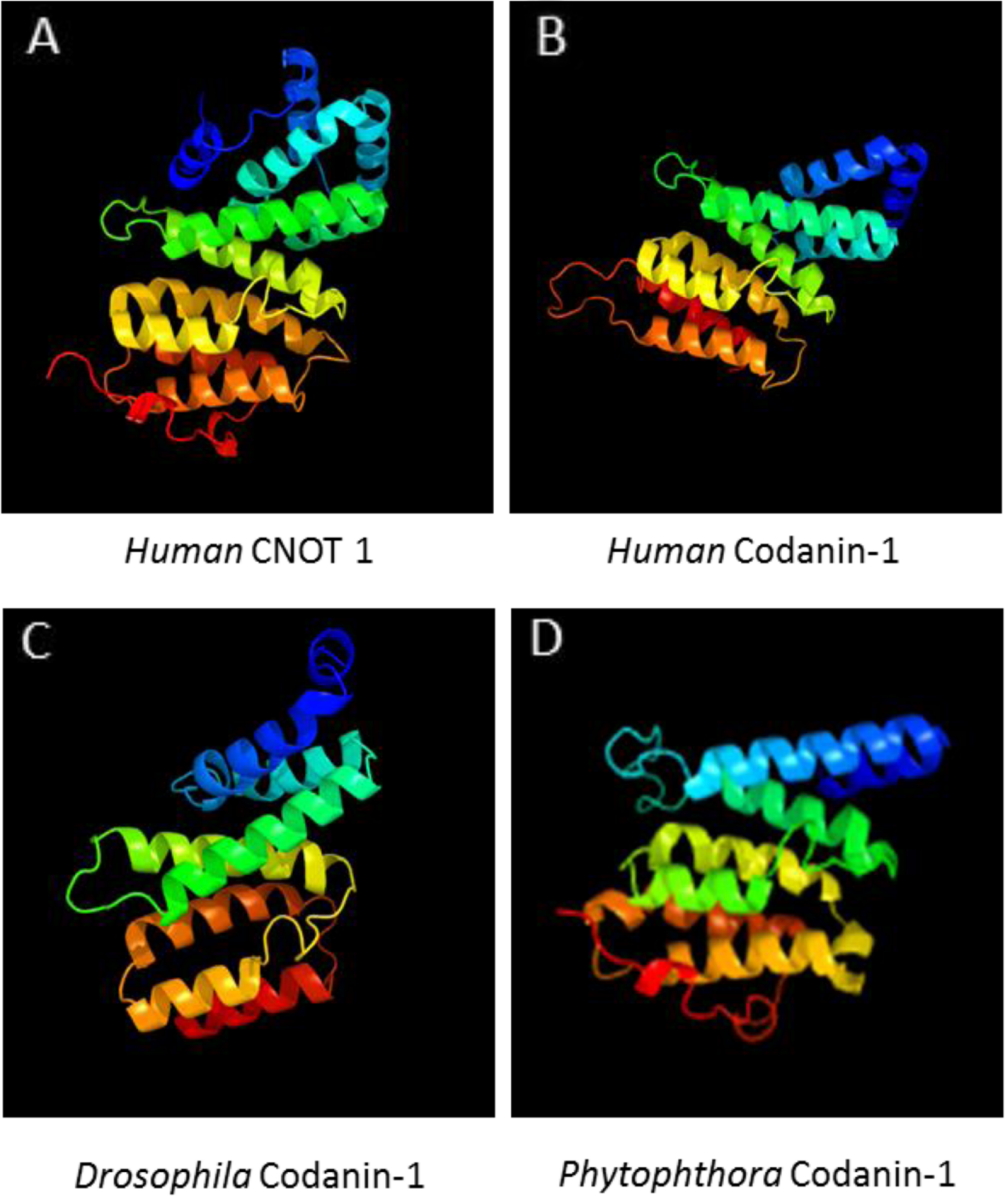
The predicted structure of the MIF4G-like domain of Codanin-1 is highly similar to the MIF4G domain in CNOT1. **a**. The known structure of the MIF4G domain of the human CNOT1 (a.a.1103 –1287) (PDB 4CT4; [19]). **b**-**d**. Predicted structures of the parallel regions of Codanin-1 in human, *Drosophila* and *Phytophthora*, based on the CNOT1 template shown in (**a**)

The three domains are arranged in the same order and with similar spacing in Codanin-1 and CNOT1 (Fig. 6b). Notably, the region giving the highest confidence score (a.a. 478–758 in human Codanin-1) is included in the region which is highly conserved through the evolution of the codanin-1 proteins (~a.a. 400–850). The 3D structural similarity of Codanin-1 to CNOT1 is conserved and was found in all Codanin-1-like proteins examined. For example, the two highest scores for modeling *Drosophila melanogaster* Codanin-1 homolog, discs lost, were found in CNOT1 (similarity of 96.7% confidence to a.a. 1111–1265 and 56.1% confidence to a.a. 895–988 of human CNOT1). Similarly, the oomycetes fungus-like *Phytophthora parasitica* Codanin-1 homolog (protein F441_15879; 1340 a.a.) has similarity of 98.9% confidence to a.a. 1352–1464 and of 98.8% confidence to a.a. 1143–1309 of human CNOT1. The predicted model of the most conserved codanin-1 domain in human, *Drosophila* and *Phytophtora* as well as the corresponding domain in the human CNOT1 template are seen in Fig. 7.

CNOT1 is the large, scaffolding subunit of the conserved CCR4–NOT complex, which is mainly involved in RNA-related processes including mRNA deadenylation, translational repression and transcriptional control [17, 20]. The deadenylation is performed by the CAF1 and CCR4A exoribonuclease, which are components of the CCR4–NOT complex. The interactions between CNOT1 and its binding proteins were mapped in yeast, human and *Drosophila* [19, 21–23]. Interestingly, the two most conserved regions between Codanin-1 and CNOT1 are mapped to well documented CNOT1 domains. A central region in CNOT1 (spanning a.a. 1152–1376 in *Drosophila* CNOT1), designated NOT1 MIF4G domain, has been shown to bind the deadenylase CAF1 [23]. The CAF1 deadenylase binds the CCR4A deadenylase and thus bridges between CNOT1 and CCR4A [21]. NOT1 MIF4G domain has also been shown to bind to the DEAD-box protein DDX6, which functions as a translational repressor and decapping activator [19, 24]. The MIF4G domain is the region which served to model Codanin-1 with the highest confidence (97.8%)(Fig. 6b). The second highest similar region (89.4% confidence), spanning a.a. 1381–1535 in human CNOT1, is designated CAF40/NOT9-binding domain (CN9BD). The CN9BD domain is also implicated in mRNA metabolism and function: it binds CAF40, a scaffold protein for several proteins which are involved in miRNA degradation [19], degradation of mRNAs containing AU-rich elements [25], and mRNA decay and translational repression [26].

CNOT1 has been demonstrated to bind and repress ligand-dependent transcriptional activation by estrogen receptor α. The binding and repression are dependent on nine LXXLL motifs (where L is leucine and X any amino acid; termed nuclear receptor boxes) present in CNOT1 [27]. Intriguingly, eight LXXLL motifs exist in human Codanin-1. To examine the statistical probability for a random existence of this motif in Codanin-1, we performed a protein sequence simulation in which the Codanin-1 sequence was shuffled 10,000 times (i.e. keeping the single amino acids frequencies). In only 194 cases we got the same number (8) or higher number of the LXXLL pattern. This will amount to statistical significance of *P*-Value < 0.02, strengthening the structural similarity between the two proteins, and suggesting involvement of Codanin-1 in nuclear receptors signaling and transcription.

Taken together, the structural similarity between Codanin-1 and CNOT1 suggests that Codanin-1 is involved in similar pathways, namely in transcriptional and post-transcriptional control of RNA levels and functions.

## Discussion

Mutations in C15Orf41 gene in patients were reported to result in very similar phenotypes to Codanin-1 mutations [14]. However, physical interaction between the two proteins was not described. Our co-immunoprecipitation assays revealed physical interaction between the two proteins, and localized the binding to the C-terminus of Codanin-1. Thus, it differs from the previously described interaction of the histone chaperon ASF1 with the N-terminus of Codanin-1. As codanin-1 is a big protein with no apparent enzymatic activity it probably serves as a scaffold for recruitment of ASF1, C15Orf41, HP1α [11] and probably also histone H3-H4 and importin-4 [15]. Supporting the existence of this complex is the large scale survey which report C15Orf41 binding to ASF1b [28]. Taken together, the assumption that Codanin-1’s function is to recruit several different proteins, and the clinical observation that mutations on different positions in Codanin-1 (presumably binding different proteins) results in highly similar erythroid phenotype, suggest a unified function(s) for this complex.

Overexpression of Codanin-1 results in C15Orf41 stabilization and shifted it from the nucleus to the cytoplasm. Interestingly, it has been previously reported that Codanin-1 overexpression moves ASF1 from the nucleus to the cytoplasm [15]. Thus, one presumptive function of Codanin-1/C15Orf41/ASF1 complex could be inhibition or modulation of specific activities within the nucleus. The identity of this function is not clear. However, as ASF1 is a histone chaperone operating during nucleosome assembly and disassembly, and as C15Orf41 is similar to members of the Holliday junction resolvase family, involvement of this complex in chromatin assembly is the immediate suspect.

Phylogenetic profiling is a well-supported method for predicting functional relations between proteins [16]. Analysis of Codanin-1 and C15Orf41 distribution in different organisms revealed very high correlation between the existence and elimination of the two genes. Both of the proteins were lost in five unrelated taxa: Porifera, Nematoda, Tardigrada, Plathyhelminthes and Mesozoa. This is one of the extreme cases of the phenomenon of Phylogenetic profiling, which strongly suggest common pathways. Quite counterintuitively, C15Orf41 exists in all taxa in which Codanin-1 exist, but not vice versa, suggesting that the major function of the presumptive scaffold protein, Codanin-1, is to regulate C15Orf41 activities. Additional support for common functions of these two proteins, is the recent report documenting a correlation between the levels of the two genes in different cell lines [29]. The characterization of these functions and their relation to erythrocyte developments should await further investigation.

Lastly, even though codanin-1 is usually reported as an orphan protein with no apparent significantly related protein, we found that its predicted spatial structure is highly similar to the structure of CNOT1. Importantly, the Codanin-1 domains which display similarity to CNOT1 are those which are most highly conserved during evolution. The similar 3D structure between Codanin-1 and CNOT1 suggests common ancestral protein, similar scaffolding function for Codanin-1, and possibly involvement in similar processes. The CCR4–NOT complex has multi tasks and thus predicting which functions are conserved in Codanin-1 (and its binding proteins) is not a trivial mission. However, as the highest confident similarities are localized within the MIF4G and CN9BD domains, both of which are involved in mRNA (or miRNA) stability and in translational repression [30], it suggests involvement of Codanin-1 complex in RNA-related functions.

## Conclusions

The current study demonstrates intimate evolutionary connection between Codanin-1 and C15Orf41, and suggest that Codanin-1 serves as a scaffold protein that stabilizes and localizes C15Orf51 within the cytosol. The high resemblance between the 3D predicted structure of Codanin-1 and the scaffold protein CNOT1 suggests a common ancestor and common roles for these two proteins, namely involvement in RNA metabolism and function. Further studies will be needed to reveal whether the interaction between Codanin-1 and C15Orf41 or ASF1 is functionally related to the presumable CNOT1-like activities of Codanin-1.

## Methods

### Cell culture, transfections and co-immunoprecipitation

HeLa cells were grown in DMEM - high-glucose (Gibco) supplemented with 10% heat-inactivated fetal bovine serum (FBS), 100 IU/ml penicillin, and 100 μg/ml streptomycin at 37 °C in a 5% CO2 atmosphere. Transfections were conducted using the Polyethyleneimine (PEI) reagent. 2 h before transfection the medium was changed to DMEM + 2% FCS. For transfection of 10 cm plate, 10 μg of DNA (unless specified otherwise) and 45 μl of 1 μg/μl PEI were added to 1 ml of Opti-MEM (Gibco). The solution was mixed by Vortex, incubated at R.T. for 15 min and added to the cells. Several of the experiments were also performed using Metafectene (Biontex Laboratories) according to the manufacturer’s recommendations, yielding similar results. For quantification of the half-life of the proteins, the cells were treated for the indicated periods with 25 μg/ml of the translational elongation inhibitor, cycloheximide. Inhibition of the proteasome was performed using MG132 at final concentration of 20 mM for 5 h. For protein extraction, cells were washed in PBS and lysed in lysis buffer (50 mM Tris/HCl pH 7.4; 100 mM NaCl; 20 mM NaF; 1 mM EDTA; 1 mM EGTA; 1% TX-100; 1 mM DTT) supplemented with protease inhibitors (1 μg/ml Leupeptin; 0.5 mM PMSF; 1 mg/ml Aprotinin) and phosphatase inhibitors (2 mM Na3VO4; 25 nM Calyculin A; 10 mM β-Glycerophosphate) .

Cells were mopped with a rubber cell scraper and the suspension was incubated on ice for 10 min. To precipitate cell remnants, the suspensions were centrifuged at 4 °C for 10 min at 17,000 g. Protein concentration was obtained by Bradford analysis (BIO-RAD), and 20 μg were used for each lane. The samples were subjected to 10% SDS-polyacrylamide gel electrophoresis, and blotted onto 0.2-μm cellulose nitrate membranes (NUPORE, India). Membranes were rotated overnight at 4 °C with primary antibodies in TBST (20 mM Tris pH = 7.5, 150mN NaCl, 0.1% v/v Triton-X 100) and 5% skim milk. Detection was performed with the following primary antibodies: codanin-1 (Santa Cruz – sc-365,839), α-tubulin (DSHB, IowaCity, 12G10), ASF1b (Cell Signaling Technologies, #2902) GFP (DSHB, 12A6), HA (Biolegend, # BLG-901501), P53 (Santa Cuz, sc-126), Flag (Genescript – A00187), A Lamin (DSHB 4A7) and β-Catenin (BD Transduction Laboratories, San Jose, CA, # 610154). Following washes, the membranes were incubated for 1 h at room temperature with the matching secondary antibody conjugated to horse radish peroxidase (HRP) (Goat anti-mouse and donkey anti-rabbit secondary antibodies in 1:20,000 dilution with PBST; Jackson ImmunoResearch, West Grove, PA). Signals were detected by ECL (Millipore) on a LAS4000 system (GE Healthcare).

For immunoprecipitation experiments, cell lysates were centrifuged for 10 min at 14,000 rpm and equivalent amounts of protein, as determined by Bradford assay, were used for immunoprecipitations. 30 μl of 50% protein A-sepharose slurry beads (Santa Cruz biotechnology) were incubated with the primary antibodies for 4 h’ at 4 °C. In parallel, 600 μg of the cell lysates were precleared with 15 μl of protein A slurry, for 1 h. The cleared lysate was incubated with the Protein A agarose- antibody suspension at 4 °C for 4 h. After extensive washes with 1XPBS, the immunoprecipitates were eluted by boiling for 5 min in SDS sample buffer and subjected to MS/MS analysis or to SDS-PAGE and Western blotting. At least three biological replicates were performed for each experiment. The MS/MS analysis was performed in the Smoler Proteomics Center, Technion, Haifa, Israel.

### Subcellular fractionation

Due to the short half-life of C15Orf41 the REAP (Rapid, Efficient And Practical) protocol was used [31]. To verify even loading and purity of the extracts, tubulin and A lamin were used as cytosolic and nuclear markers, respectively.

### Immunofluorescence

For immunofluorescence microscopy, cultures grown on coverslips were fixed in 4% paraformaldehyde for 20 min, blocked with 20% FBS/0.5% Triton X-100 in PBS, and incubated overnight with primary antibodies at 4 °C. Primary antibodies were as indicated above with one exception: the anti-HA antibodies were from arigo Biolaboratories (ARG55095). Secondary fluorescein isothiocyanate (FITC)-conjugated and rhodamin conjugated antibodies were purchased from Jackson ImmunoResearch. Staining was analyzed using an AxioImager microscope (Zeiss). The C15Orf41 and Codanin-1 staining within the cell images were quantified with ImageJ software (NIH). The nucleus or cytoplasmic area were selected by the freehand selections tool, followed by the ‘Measure’ command. Several cells (11–15) were analyzed per treatment. Averaged background of Intense Density (area x mean) from un-transfected cells was subtracted from each one of the cells’ intensities.

### Phylogenetic analysis

The protein sequences of human Codanin-**1 (**Q8IWY9–2) and C15Orf41 (Q9Y2V0–1) were used to conduct BLASTs searches against the protein sequences of each of the indicated taxons. The E values in all of the searches were found to be either clearly positive, namely E value lower that 10^− 12^, or clearly negative (E value higher than 1). In general, the E values were as expected form the phylogenetic tree (in the case of existence of a homologue), with one noted exception: The E value of the relatedness between human and Choanoflagellate C15Orf41 was 10^− 12^, while the E value between human and Archaeal C15Orf41 was 10^− 34^ (not shown). The phylogenetic tree was constructed according to the literature (e.g. The Taxonomy browser at the NCBI). As the location of the Mesozoa on the phylogenetic tree is highly debated, we followed the recent analyses placing this (polyphyletic) group within the Lophotrochozoa [32].

### Modelling the three-dimensional structure of Codanin-1

Template search and the modelling of the three-dimensional structure of human Codanin-1 was performed using the Protein Homology/analogy Recognition Engine V2.0 (Phyre2) software. The “intensive” mode, and the default parameters of the program were used.

## Supplementary information

**Additional file 1:Figure S1.** Codanin-1 enhancement of C15Orf41 levels is not dependent on co-transfection. A. Tet-On cells stably transfected with TRE-Codanin-1 were transiently transfected with C15Orf41 or as a control with a GFP construct. 24 h’ later the cells were incubated with doxycycline (or the solvent) for another 24 h’ to induce Codanin-1 expression. B. Tet-Off cells stably transfected with TRE-C15Orf41 were grown in the presence of doxycycline. The cells were transiently transfected with Codanin-1 and 24 h’ later induction of C15Orf41 was achieved by withdrawing doxycycline from the medium for 24 h’. Control cells were grown in the presence of doxycycline. Western blots were incubated with the indicated antibodies. As loading control β-Tubulin was used.

**Additional file 2:Figure S2.** MG132 lowers C15orf41 levels in cells co-expressing Codanin-1. HeLa cells were either not transfected or transfected with the indicated constructs. The transfected cells were incubated for five hours with MG132 or with the solvent, DMSO. Western blots were incubated with antibodies against Codanin-1 and C15Orf41. As loading control β-Tubulin was used. The activity of MG132 was assessed by antibodies against the endogenous β-Catenin protein. In the C15Orf41 panel, the lower band is probably not specific.

**Additional file 3:Figure S3.** Cellular localization of HA-C15orf41 and Flag-Codanin-1. HeLa cells were co-transfected with HA-C15orf41 and either Codanin-1-Flag Fragment 1, 2, 3 and 6 then reacted against HA (green) and Flag (red) antibodies. Immunofluorescence visualization of cells was performed with axioimager microscopy.

**Additional file 4:Figure S4.** Cellular localization of ASF1a co-transfected with Codanin-1 sub-fragments. HeLa cells were transfected with Flag-Codanin-1, fragment 1–3, or R1042W Codanin-1 and then reacted against ASF1a (green) and Flag (red) antibodies. Immunofluorescence visualization of cells was performed with axioimager microscopy.

## Data Availability

The datasets used and/or analysed during the current study are available from the corresponding author on request.

## Abbreviations

a.a: Amino acid
Cas9/sgRNA: CRISPR-associated nuclease 9/ single guide RNA
CDA: Congenital dyserythropoietic anemia
CHX: Cycloheximide
CN9BD: NOT9-binding domain
CNOT1: Negative Regulator Of Transcription 1
DMEM: Dulbecco′s modified - Eagle′s medium
dpc: days post coitum
miRNA: microRNA
SDS-PAGE: Sodium dodecyl sulfate–polyacrylamide gel electrophoresis
TRE: Tet-responsive element
