# Supplementary material for: Characterization of the interactions between Codanin-1 and C15Orf41, two proteins implicated in congenital dyserythropoietic anemia type I disease
Source: BMC Mol Cell Biol. 2020 Mar 23;21:18. doi: 10.1186/s12860-020-00258-1 (PMC7092493; doi:10.1186/s12860-020-00258-1)
Supplement: Supplementary file 1 — Additional file 1:Figure S1. Codanin-1 enhancement of C15Orf41 levels is not dependent on co-transfection. A. Tet-On cells stably transfected with TRE-Codanin-1 were transiently transfected with C15Orf41 or as a control with a GFP construct. 24 h’ later the cells were incubated with doxycycline (or the solvent) for another 24 h’ to induce Codanin-1 expression. B. Tet-Off cells stably transfected with TRE-C15Orf41 were grown in the presence of doxycycline. The cells were transiently transfected with Codanin-1 and 24 h’ later induction of C15Orf41 was achieved by withdrawing doxycycline from the medium for 24 h’. Control cells were grown in the presence of doxycycline. Western blots were incubated with the indicated antibodies. As loading control β-Tubulin was used. [file 12860_2020_258_MOESM1_ESM.docx]

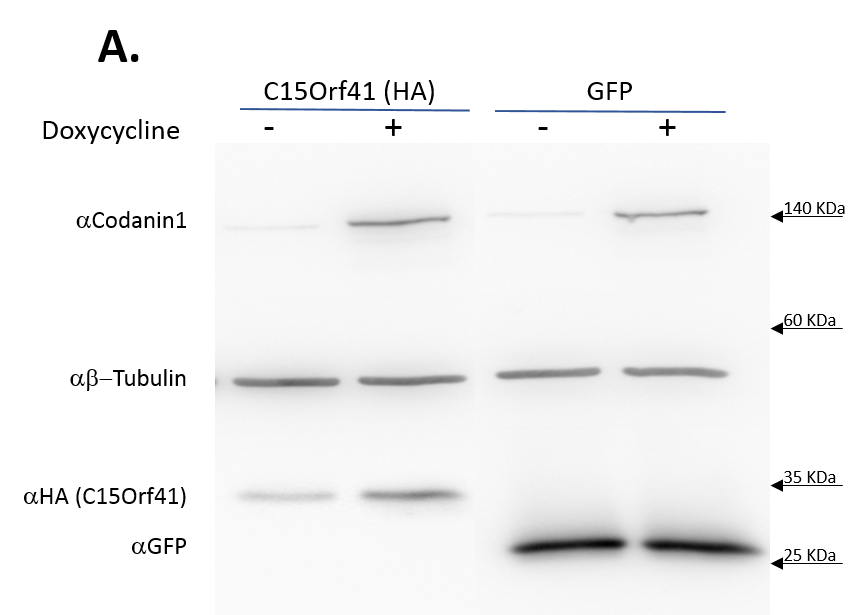


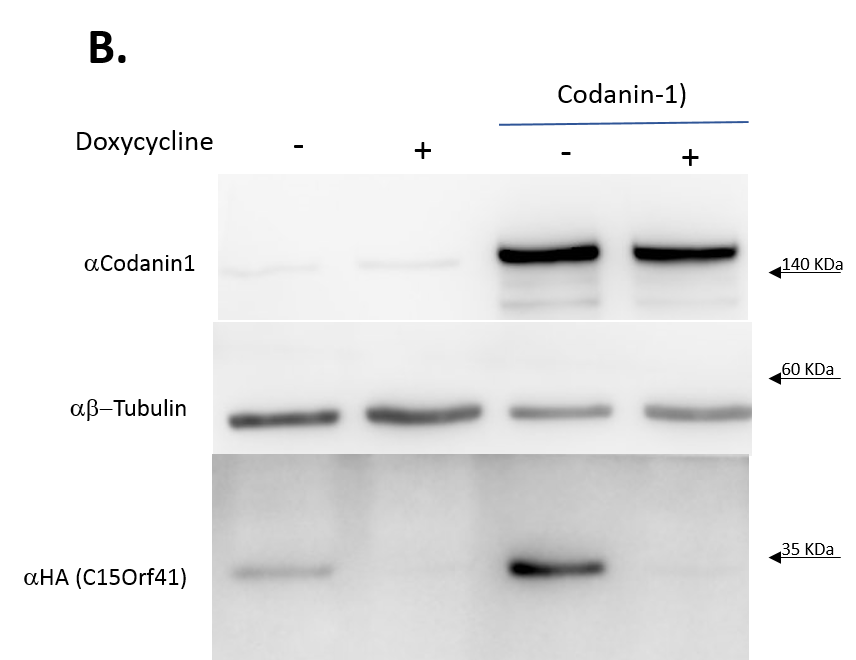


**Supplementary Figure 1. Codanin-1 enhancement of C15Orf41 levels is not dependent on co-transfection. A.** Tet-On cells stably transfected with TRE-Codanin-1 were transiently transfected with C15Orf41 or as a control with a GFP construct. 24 h’ later the cells were incubated with doxycycline (or the solvent) for another 24 h’ to induce Codanin-1 expression. **B**. Tet-Off cells stably transfected with TRE-C15Orf41 were grown in the presence of doxycycline. The cells were transiently transfected with Codanin-1 and 24 h’ later induction of C15Orf41 was achieved by withdrawing doxycycline from the medium for 24 h’. Control cells were grown in the presence of doxycycline. Western blots were incubated with the indicated antibodies. As loading control β-Tubulin was used.
