# Supplementary material for: Characterization of the interactions between Codanin-1 and C15Orf41, two proteins implicated in congenital dyserythropoietic anemia type I disease
Source: BMC Mol Cell Biol. 2020 Mar 23;21:18. doi: 10.1186/s12860-020-00258-1 (PMC7092493; doi:10.1186/s12860-020-00258-1)
Supplement: Supplementary file 2 — Additional file 2:Figure S2. MG132 lowers C15orf41 levels in cells co-expressing Codanin-1. HeLa cells were either not transfected or transfected with the indicated constructs. The transfected cells were incubated for five hours with MG132 or with the solvent, DMSO. Western blots were incubated with antibodies against Codanin-1 and C15Orf41. As loading control β-Tubulin was used. The activity of MG132 was assessed by antibodies against the endogenous β-Catenin protein. In the C15Orf41 panel, the lower band is probably not specific. [file 12860_2020_258_MOESM2_ESM.docx]

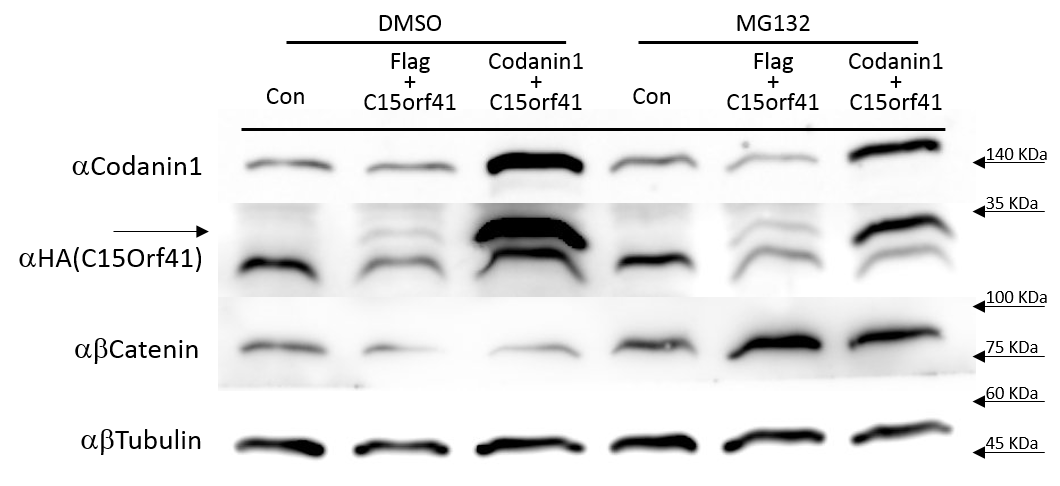


**Supplementary Figure 2. MG132 lowers C15orf41 levels in cells co-expressing Codanin-1.** HeLa cells were either not transfected or transfected with the indicated constructs. The transfected cells were incubated for five hours with MG132 or with the solvent, DMSO. Western blots were incubated with antibodies against Codanin-1 and C15Orf41. As loading control β-Tubulin was used. The activity of MG132 was assessed by antibodies against the endogenous β-Catenin protein. In the C15Orf41 panel, the lower band is probably not specific.
