# Supplementary material for: Characterization of the interactions between Codanin-1 and C15Orf41, two proteins implicated in congenital dyserythropoietic anemia type I disease
Source: BMC Mol Cell Biol. 2020 Mar 23;21:18. doi: 10.1186/s12860-020-00258-1 (PMC7092493; doi:10.1186/s12860-020-00258-1)
Supplement: Supplementary file 3 — Additional file 3:Figure S3. Cellular localization of HA-C15orf41 and Flag-Codanin-1. HeLa cells were co-transfected with HA-C15orf41 and either Codanin-1-Flag Fragment 1, 2, 3 and 6 then reacted against HA (green) and Flag (red) antibodies. Immunofluorescence visualization of cells was performed with axioimager microscopy. [file 12860_2020_258_MOESM3_ESM.docx]

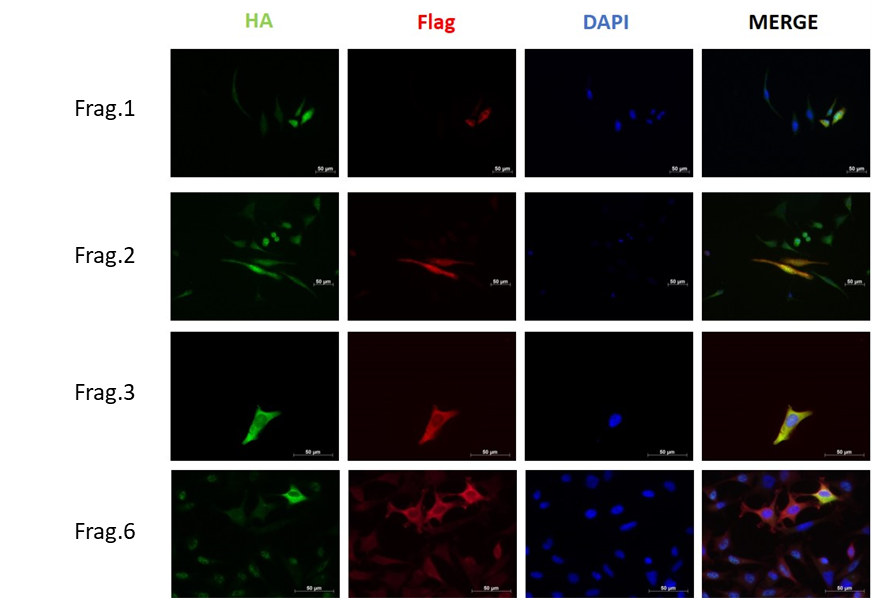


**Supplementary Fig. 3. Cellular localization of HA-C15orf41 and Flag-Codanin-1.** HeLa cells were co-transfected with HA-C15orf41 and either Codanin-1-Flag Fragment1, 2, 3 and 6 then reacted against HA (green) and Flag (red) antibodies. Immunofluorescence visualization of cells was performed with axioimager microscopy.
