# Supplementary material for: Characterization of the interactions between Codanin-1 and C15Orf41, two proteins implicated in congenital dyserythropoietic anemia type I disease
Source: BMC Mol Cell Biol. 2020 Mar 23;21:18. doi: 10.1186/s12860-020-00258-1 (PMC7092493; doi:10.1186/s12860-020-00258-1)
Supplement: Supplementary file 4 — Additional file 4:Figure S4. Cellular localization of ASF1a co-transfected with Codanin-1 sub-fragments. HeLa cells were transfected with Flag-Codanin-1, fragment 1–3, or R1042W Codanin-1 and then reacted against ASF1a (green) and Flag (red) antibodies. Immunofluorescence visualization of cells was performed with axioimager microscopy. [file 12860_2020_258_MOESM4_ESM.docx]

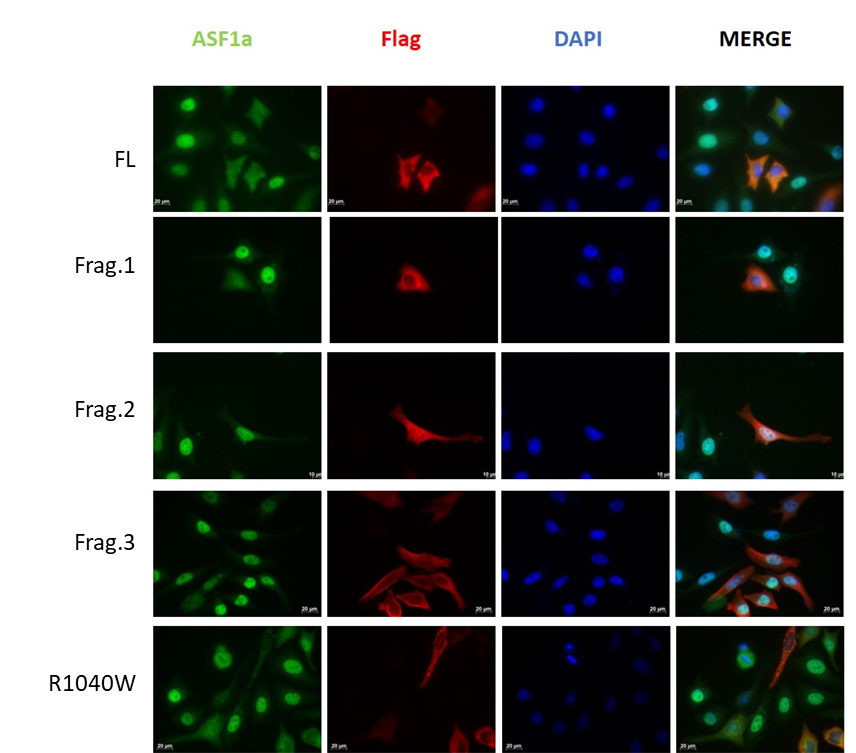


**Supplementary Fig. 4. Cellular localization of ASF1a co-transfected with Codanin-1 sub-fragments.** HeLa cells were transfected with Flag-Codanin-1, fragment 1-3, or R1042W Codanin-1 and then reacted against ASF1a (green) and Flag (red) antibodies. Immunofluorescence visualization of cells was performed with axioimager microscopy.
